# Inhibition of autophagy enhances apoptosis induced by proteasome inhibitor bortezomib in human glioblastoma U87 and U251 cells

**DOI:** 10.1007/s11010-013-1835-z

**Published:** 2013-10-09

**Authors:** Xudong Zhang, Weiming Li, Chunlan Wang, Xiangyang Leng, Shulin Lian, Jingbin Feng, Jinliang Li, Hailiang Wang

**Affiliations:** 1 Affiliated Hospital of Changchun University of Traditional Chinese Medicine, Changchun, 130033 Jilin China; 2 Jilin Academy of Traditional Chinese Medicine Hospital of Jilin Province, Changchun, 130033 Jilin China; 3 The People’s Hospital of Sanya City Hainan Province, Sanya, 572000 Hainan China; 4 The Yong Plastic Surgery Clinic, Shuangliao, 136200 Jilin China; 5 The Second Clinical Hospital of Jilin University, Changchun, 130033 Jilin China

**Keywords:** Glioblastoma, Autophagy, Apoptosis, Bortezomib

## Abstract

Glioblastoma is the most aggressive cerebral gliomas. Despite advances in therapies, the prognosis is still very poor. Therefore, novel therapeutic strategies are required. As a proteasome inhibitor, bortezomib has shown its efficacy as an active antitumor agent against a variety of tumors. However, inhibition of proteasome activity leads to cell death and also induces cell autophagy, and due to the dual roles of autophagy in the survival and death of tumor cells, the effect of inhibition of autophagy on glioblastoma cells remains to be explored. We therefore assessed whether bortezomib is capable of inducing autophagy, and investigated the antitumor effect of bortezomib combined with autophagy inhibitors on human glioblastoma U251 and U87 cells. Cell viability was measured by MTT assay. The expressions of autophagy and apoptosis-related proteins were determined by Western blot analysis. U251 and U87 cells proliferation was inhibited in a dose-dependent manner. Both apoptosis and autophagy induced by bortezomib were observed in human glioblastoma U87 and U251 cells. However, when U251 and U87 cells were co-treated with bortezomib and autophagy inhibitors 3-MA or Atg7 siRNA, the autophagy inhibitors blocked the autophagy in the cells and resulted in a further inhibition of cell proliferation and a further increase in cell apoptosis as compared with that treated with bortezomib alone. These findings indicated that combination of bortezomib and autophagy inhibitors may shed new light on glioblastoma treatment.

## Introduction

Glioblastoma is the most aggressive and the most frequent common tumor, comprising approximately 50 % of the cerebral gliomas [1, 2]. Surgical removal of the tumor is the first-line therapy. Unfortunately, these tumor cells are highly mobile and usually infiltrate the surrounding tissues. Therefore, surgery has to be followed by chemotherapy and radiation therapy to further reduce the number of remaining tumor cells. Despite recent advances in all these therapies [3], the response is still very poor, and the median survival time for patients with glioblastoma remains about 12 months [4–6]. Therefore, novel therapeutic strategies are required.

One emerging treatment strategy in glioblastoma involves novel agents such as proteasome inhibitors [7]. Preclinical studies illustrated a broad antitumor activity of bortezomib [8]. Bortezomib functions as a selective inhibitor of the 26S proteasome, producing predictable, dose-related, and reversible proteasome inhibition. It has shown antitumor activity in a variety of malignancies and also was the first proteasome inhibitor used in clinical practice [9].

Although bortezomib is now approved for the treatment of multiple myeloma, numerous clinical trials with bortezomib have shown its efficacy as an active antitumor agent against a variety of solid tumors [10]. Several researches demonstrated that bortezomib is relatively well tolerated, resulting in manageable nonhaematological and haematological toxicity. Clinical studies showed high response rates in refractory multiple myeloma patients and good tolerance to bortezomib [11, 12]. It was applied as a single agent and in combination with other chemotherapeutic drugs, showing potent effect. In a variety of other haematological malignancies and solid tumors, clinical phase I and II studies using bortezomib alone or together with other drugs have showed encouraging results, both in children and adults [13–18], for many carcinomas. However, the progress of researches about the effect of bortezomib on glioblastoma is quite limited.

Resistant to apoptosis induced by chemotherapy is one of the most important features of tumor cells, and also contributes to drug fast, tumor recurrence, and metastasis. Some researches revealed that as one of the protective mechanism in cells, activation of the autophagy pathway may play an important role in apoptosis resistance.

Autophagy is an evolutionarily conserved, intracellular self-defense mechanism characterized by the formation of double-membraned autophagic vesicles, in which long-lived, aggregated, misfolded proteins, and damaged organelles are sequestered and subsequently degraded through fusion with lysosomes. In general, autophagy functions to maintain cellular homeostasis through nutrition recycling and protein quality control [19]. In the context of diseases, autophagy has been seen as an adaptive response to survival, whereas in other cases it appears to promote cell death and morbidity [20]. Increasing evidence indicates that autophagy may be activated during chemotherapies in cancer cell lines such as breast cancer cell MCF-7 and colon cancer cell HCT116 [21]. Recent studies on the role of autophagy have highlighted the advances in the pharmacologic manipulation of autophagy pathways as a therapeutic strategy for cancer [22, 23]. However, whether such autophagy contributes to tumor cell death or is a mechanism of resistance remains uncertain and may vary depending on stimulus type, nutrient availability, organism development, and apoptotic status [24].

Based on recent studies [25, 26], it was found that bortezomib may have growth inhibition on glioblastoma cells. So we chose two glioblastoma cell lines U251 and U87 to explore the ability of bortezomib on apoptosis and autophagy in glioblastoma cell lines. Furthermore, we also detected whether inhibition of autophagy would enhance the cell apoptosis rate when bortezomib was used.

## Materials and methods

### Materials

Bortezomib was purchased from Chemie Tek. Atg7 siRNA plasmid was purchased from the Santa Cruz Biotechnology (sc-41447). Fetal bovine serum (FBS) and Dulbecco’s modified eagle media (DMEM) were purchased from GIBCO. 3-(4,5-dimethylthiazol-2-yl)-2,5-diphenyltetrazolium bromide (MTT), 3-methyladenine (3-MA), and Bafilomycin A1 were purchased from Sigma. The antibodies anti-LC3, anti-Beclin 1, anti-caspase-3, anti-cleaved caspase-3, anti-PARP, anti-Atg7, anti-cytochrome C, anti-CoxIV, anti-Bax, and anti-Bcl-2 were purchased from Santa Cruz Biotechnology.

### Cell culture

The human U87 and U251 cells were cultured in DMEM with 10 % FBS, under standard culture conditions (37 °C and 5 % CO_2_).

### Cell viability assays

Each group was repeated in six separate wells. U87 and U251 cells were cultured in 96-well plates at a density of 5 × 10^3^ cells/well in 150 μl of complete medium. MTT reagent (10 μl, 5 mg/ml) was added to each well for another 4 h. After treatment with MTT each well was dissolved in 120 μl DMSO. Absorbance was recorded at a wavelength of 490 nm.

### Flow cytometry analysis

In brief, after treatment with bortezomib, U87 and U251 cells were incubated at 37 °C for 20 min with propidium iodide (PI, 1 mg/ml, Invitrogen) and Annexin V-FITC (1 mg/ml, Invitrogen) to determine the number of apoptotic cells. Then, the samples were analyzed by a FACScan flow cytometer (Becton–Dickinson, Franklin Lakes, NJ).

### Cytochrome c release assay

After treatment with bortezomib, U87 and U251 cells were harvested in a hypotonic solution [10 mM HEPES (pH 7.9), 0.1 mM EDTA, 10 mM KCl, 1 mM DTT, and 0.5 mM PMSF], and incubated in cold room for 20 min. Cell lysates were centrifuged at 700×*g* for 5 min and then the pellet was removed . For the mitochondrial fraction, the supernatant was centrifuged at 10,000×*g* for 20 min. The supernatant was used as crude cytosolic and pellet was used as mitochondrial fractions. The mitochondrial pellets and corresponding supernatants were used for immunoblot analysis.

### Atg7 siRNA transfection

For transfection, about 50 % U87 and U251 cells were grown in each dish. And then, these cells were transfected with 60 nmol/l of siRNA Atg7 using Lipofectamine RNAiMAX (Invitrogen, Carlsbad, CA) according to the manufacturer’s protocol. U87 and U251 cells were harvested for western blot at 30 h posttransfection.

### Mitochondrial membrane potential analysis

We used the JC-1 staining (Invitrogen Life Technologies, Carlsbad, CA, USA) through flow cytometry to detect the change of mitochondrial membrane potential (MMP) in U87 and U251 cells. The assay was performed according to the manufacturer’s protocol. U87 and U251 cells were washed with PBS for three times and resuspended in PBS at a concentration of 1–2 × 10^6^ cells/ml. And then U87 and U251 cells were stained with 4 μl of JC-1 (1 mg/ml) and incubated in the darkroom at 37 °C for 1.5 h. The JC-1 positive U87 and U251 cells were subsequently detected by FACSCalibur flow cytometer.

### Western blot

After treatment with bortezomib alone or together with autophagic inhibitor 3-MA, U87 and U251 cells were washed with cold PBS twice and then 220 μl radioimmunoprecipitation (RIPA) buffer (150 mM NaCl, 1 mM EDTA, 0.1 mM Na3VO4, 50 mM Tris–HCl (pH 6.8), 0.1 % SDS, 1 mM sodium fluoride [NaF], 1 % Triton X-100, 1 % NP40, 1 μg/ml aprotinin, 1 μg/ml leupeptin, 1 μg/ml pepstatin A,1 mM dithiothreitol, and 1 mM PMSF) was added to each dish. After that, U87 and U251 cells lysates were shaken in cold room (4 °C) for 15 min. Cell lysates were centrifuged at 10,000×*g* for 15 min, and protein concentrations in the supernatants were detected using the BCA Protein assay. 45 μg proteins were used for western blot analysis. These proteins were separated by 10 % (w/v) SDS–polyacrylamide gel electrophoresis. After running the gels (100 V, 1.5 h), proteins were transferred onto PVDF membrane. And then, the membrane was blocked with 5 % (w/v) skim milk in buffer (100 mM NaCl, 10 mM Tris–HCl [pH 7.6], and 0.1 % (v/v) Tween 20) for 20 mim at room temperature (25 °C) and the primary antibodies were added overnight on the shaker in cold room. The second day, PVDF membranes were incubated with secondary antibodies (Sigma) for 1 h at room temperature. The semi-quantitation of proteins was surveyed with a Tanon GIS gel imager system.

### Statistical analysis

Data are representative of three independent experiments performed in triplicate. *P* < 0.05 and *P* < 0.01 were considered to represent a statistically difference.

## Results

### Bortezomib inhibits growth and induces apoptosis through mitochondrial apoptotic pathway in human glioblastoma U251 and U87 cells

**Fig. 1:**
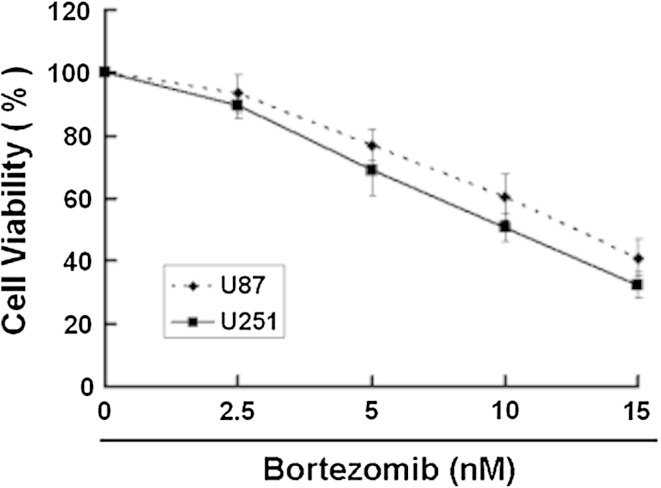
Bortezomib inhibits growth in human glioblastoma U251 and U87 cells. U251 and U87 cells were treated with 0, 2.5, 5, 10, and 15 nM bortezomib for 24 h. Cells viability was determined by MTT assay. Data are presented as mean ± SD, *n* = 6

**Fig. 2:**
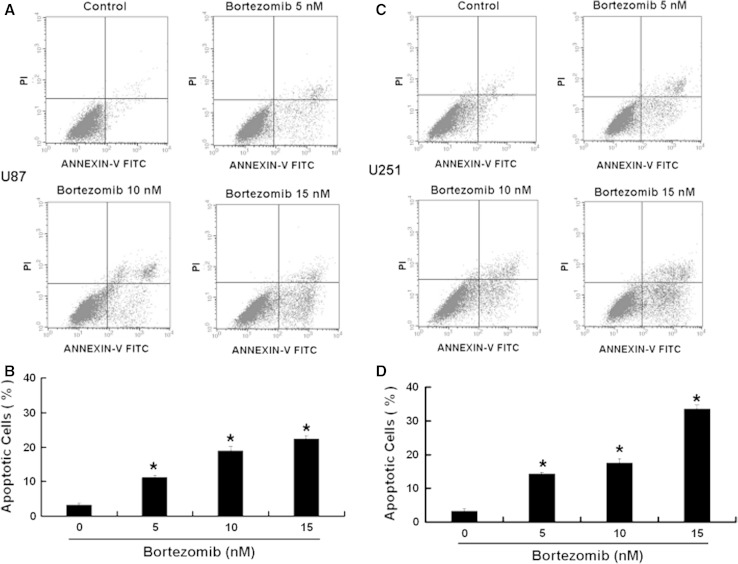
Bortezomib induces apoptosis in human glioblastoma U251 and U87 cells. **a** U87 cells were treated with 0, 5, 10, and 15 nM bortezomib for 24 h. Cells were stained with PI and Annexin V-FITC. **b** The positive stained U87 cells were counted using FACScan. **c** U251 cells were treated with 0, 5, 10, and 15 nM bortezomib for 24 h. Cells were stained with PI and Annexin V-FITC. **d** The positive stained U251 cells were counted using FACScan. Data are presented as mean ± SD, *n* = 3, *P < 0.05 versus control group

**Fig. 3:**
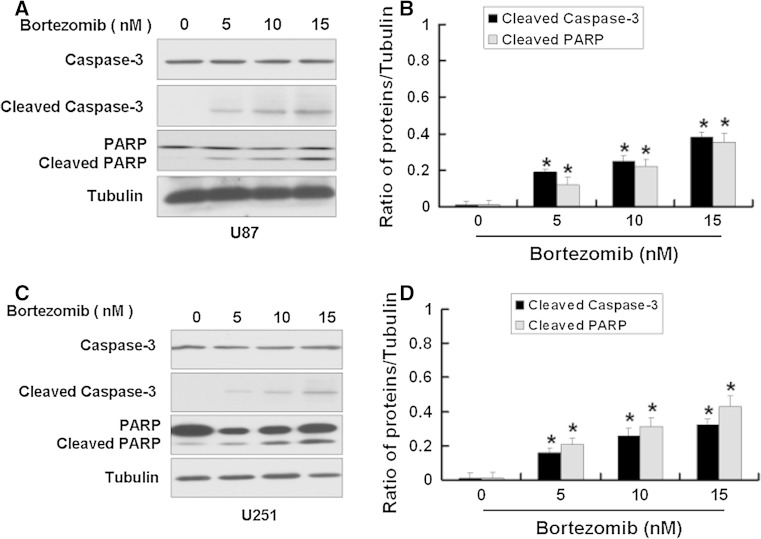
Bortezomib increased the apoptotic-related proteins in U87 and U251 cells. **a** Western blot analysis for the expressions of Caspase-3, Cleaved caspase-3, and PARP in U87 cells treated by 0, 5, 10, and 15 nM bortezomib for 24 h. **b** Quantitation of Cleaved caspase-3 and Cleaved PARP proteins levels in U87 cells. **c** Western blot analysis for the expressions of Caspase-3, Cleaved caspase-3, and PARP in U251 cells treated by 0, 5, 10, and 15 nM bortezomib for 24 h. **d** Quantitation of Cleaved caspase-3 and Cleaved PARP proteins levels in U251 cells. Data are presented as mean ± SD, *n* = 3, *P < 0.05 versus control group

**Fig. 4:**
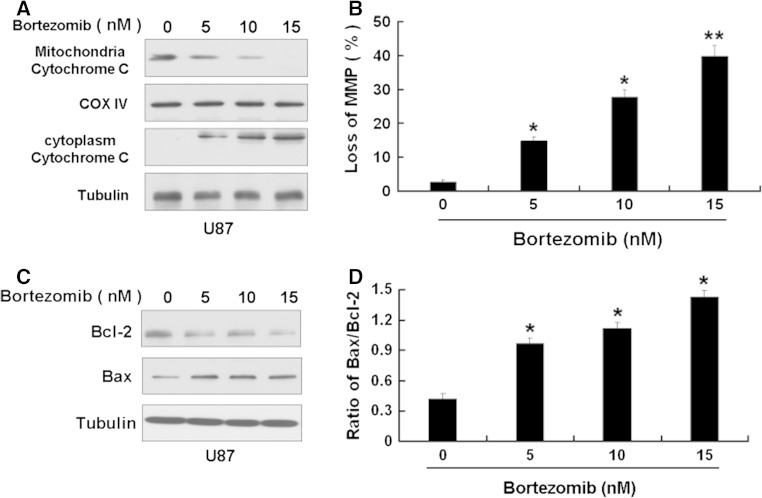
Bortezomib induces apoptosis through mitochondrial apoptotic pathway in U87 cells. **a** Western blot analysis for the expressions of mitochondria Cytochrome C and cytoplasm Cytochrome C in U87 cells treated by 0, 5, 10, and 15 nM bortezomib for 24 h. **b** U87 cells treated by 0, 5, 10, and 15 nM bortezomib for 24 h. The cells were harvested after treatment and were stained with JC-1. **c** Western blot analysis for the expressions of Bcl-2 and Bax in U87 cells treated by 0, 5, 10, and 15 nM bortezomib for 24 h. **d** Ratio of Bax/Bcl-2 in U87 cells treated by 0, 5, 10, and 15 nM bortezomib for 24 h. Data are presented as mean ± SD, *n* = 3. *P < 0.05 versus control group, **P < 0.01 versus control group

**Fig. 5:**
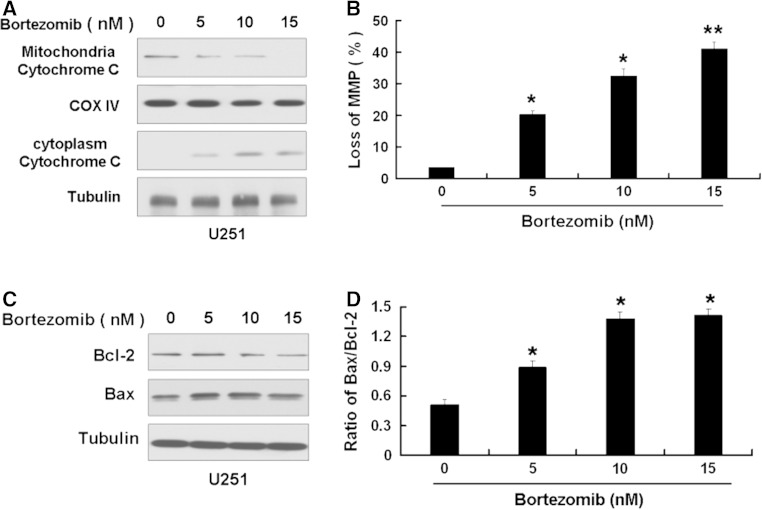
Bortezomib induces apoptosis through mitochondrial apoptotic pathway in U251 cells. **a** Western blot analysis for the expressions of mitochondria Cytochrome C and cytoplasm Cytochrome C in U251 cells treated by 0, 5, 10, and 15 nM bortezomib for 24 h. **b** U251 cells treated by 0, 5, 10, and 15 nM bortezomib for 24 h. The cells were harvested after treatment and were stained with JC-1. **c** Western blot analysis for the expressions of Bcl-2 and Bax in U251 cells treated by 0, 5, 10, and 15 nM bortezomib for 24 h. **d** Ratio of Bax/Bcl-2 in U251 cells treated by 0, 5, 10, and 15 nM bortezomib for 24 h. Data are presented as mean ± SD, *n* = 3. *P < 0.05 versus control group, **P < 0.01 versus control group

Some studies have shown that a selective inhibitor of 26S proteasome, bortezomib, has a antitumor activity [8]. So we first used MTT assay to detect the effect of bortezomib on U251 and U87 cells. We chose 3 time points 24, 48, and 72 h at the beginning. Because U251 and U87 cells died extensively at 48 h and 72 h after bortezomib treatment, so we chose 24 h for the study. As shown in Fig. 1, bortezomib reduced the cell viability of U251 and U87 cells in a dose-dependent way. Next, we wanted to know if bortezomib can induce apoptosis. First, we detected the apoptosis in U87 and U251 cells treated by bortezomib through flow cytometry. As shown in Fig. 2, bortezomib induced apoptosis in U251 and U87 cells. We further detected the apoptosis-related protein caspase-3 and PARP (poly (ADP-ribose) polymerase) in U87 and U251 cells treated by bortezomib. As seen in Fig. 3, bortezomib increased the expressions of cleaved caspase-3 and cleaved PARP in U87 and U251 cells. At the same time, we detected the expression of mitochondrial apoptotic protein Cytochrome C in U87 cells treated by bortezomib. Bortezomib increased the expression of Cytochrome C in cytoplasm and decreased the expression of Cytochrome C in mitochondria (Fig. 4a). We used JC-1 (5,50,6,60-tetrachloro-1,10,3,30-tetraethyl-benzimidazolylcarbocyanine iodide) staining to measure the MMP in U87 cells treated by bortezomib. As shown in Fig. 4b, bortezomib induced the loss of the MMP in U87 cells. Next, we also detected the expressions of mitochondrial apoptotic protein Bcl-2 and Bax in U87 cells treated by bortezomib. As shown in Fig. 4c, d, bortezomib increased the ratio of Bax/Bcl-2 in U87 cells. We also used U251 cells to confirm these results. As shown in Fig. 5, bortezomib can also induce apoptosis in U251 cells through mitochondrial apoptotic pathway.

### Bortezomib induces autophagy in U251 and U87 cells

**Fig. 6:**
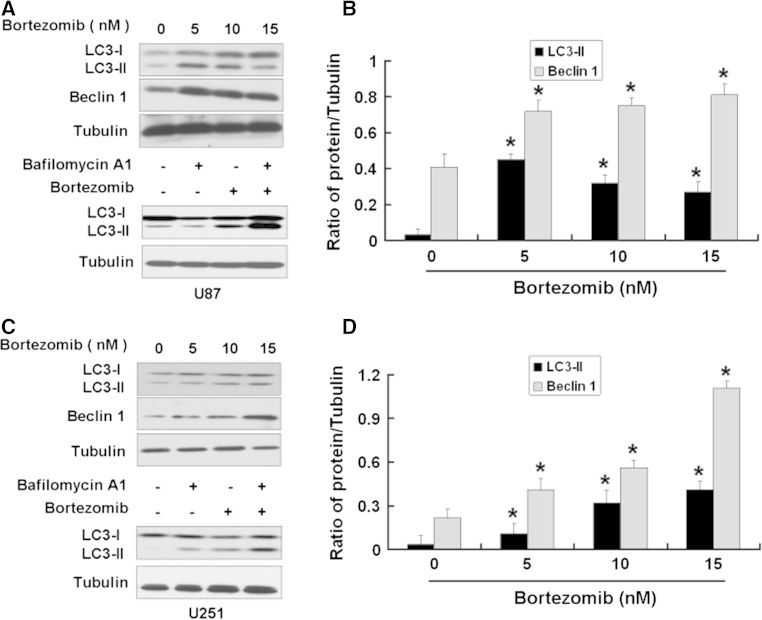
Bortezomib induces autophagy in human glioblastoma U87 and U251 cells. **a** Western blot analysis for the expressions of LC3 and Beclin 1 in U87 cells treated by 0, 5, 10, and 15 nM bortezomib for 24 h and the expression of LC3 in U87 cells treated by bortezomib (10 nM) alone or together with Bafilomycin A1 (1 μM) for 24 h. **b** Quantitation of LC3-II and Beclin 1 proteins levels in U87 cells treated by 0, 5, 10, and 15 nM bortezomib for 24 h. **c** Western blot analysis for the expression of LC3 and Beclin 1 in U251 cells treated by 0, 5, 10, and 15 nM bortezomib for 24 h and the expression of LC3 in U251 cells treated by bortezomib (10 nM) alone or together with Bafilomycin A1 (1 μM) for 24 h. **d** Quantitation of LC3-II and Beclin 1 proteins levels in U251 cells treated by 0, 5, 10, and 15 nM bortezomib for 24 h

It is reported that bortezomib can induce autophagy in tumor cells. Autophagy is an evolutionarily conserved process for degrading long-lived proteins, misfolded proteins, and damaged organelles through fusion with lysosomes. Studies have shown that autophagy plays a key role in tumor survival and apoptosis [24]. We next detected the expressions of autophagy-associated proteins LC3 and Beclin 1 in U251 and U87 cells treated with bortezomib. When autophagy occurs, microtubule-associated protein LC3 localizes to isolation membranes leading to the formation of autophagosome membranes. There are two cellular forms of LC3, LC3-I and LC3-II. LC3-I converts to LC3-II when autophagy happens and the amount of LC3-II becomes a marker for the formation of autophagosomes. As shown in Fig. 6a, b, bortezomib apparently increased the expressions of LC3-II and Beclin 1 in U87 cells and lysosome inhibitor Bafilomycin A1 can block autophagy induced by bortezomib. The similar results can be seen in another human glioblastoma cell line U251 (Fig. 6c, d). These results indicated that bortezomib could inhibit growth and induce autophagy in human glioblastoma U251 and U87 cells.

### Atg7 siRNA and 3-MA inhibit autophagy induced by bortezomib in U87 and U251 cells

**Fig. 7:**
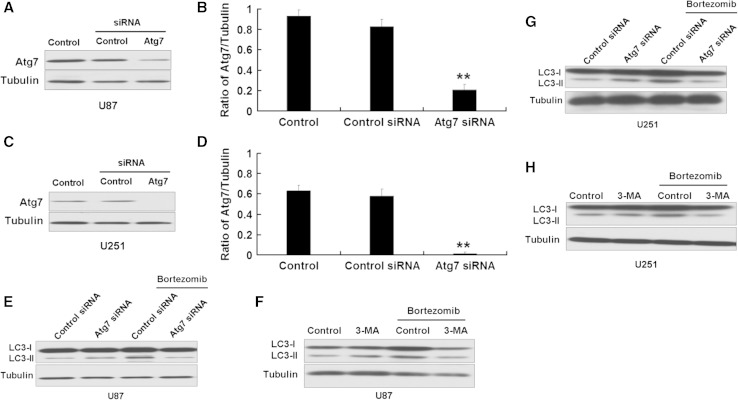
siRNA Atg7 and 3-MA inhibit autophagy induced by bortezomib in U87 and U251 cells. **a** Western blot analysis for the expression of Atg7 in U87 cells treated by control siRNA or Atg7 siRNA. **b** Quantitation of Atg7 levels in U87 cells treated by control siRNA or Atg7 siRNA. **c** Western blot analysis for the expression of Atg7 in U251 cells treated by control siRNA or Atg7 siRNA. **d** Quantitation of Atg7 levels in U251 cells treated by control siRNA or Atg7 siRNA. **e** Western blot analysis for the expression of LC3 in U87 cells treated by control siRNA or Atg7 siRNA with or without bortezomib (10 nM, 24 h). **f** Western blot analysis for the expression of LC3 in U87 cells treated by control (DMSO) or 3-MA (5 mM) with or without bortezomib (10 nM) for 24 h. **g** Western blot analysis for the expression of LC3 in U251 cells treated by control siRNA or Atg7 siRNA with or without bortezomib (10 nM, 24 h). **h** Western blot analysis for the expression of LC3 in U251 cells treated by control (DMSO) or 3-MA (5 mM) with or without bortezomib (10 nM) for 24 h. Data are presented as mean ± SD, *n* = 3, **P < 0.01 versus control group

According to the above results, bortezomib can induce both apoptosis and autophagy in U87 and U251 cells. Autophagy is always considered as a protection process when cells undergo bad conditions. However, the role of autophagy in apoptosis is uncertain. In this study, we used two methods to inhibit autophagy to explore the role of autophagy in apoptosis induced by bortezomib. ATG7 is an important component in the autophagic machinery facilitating the degradation of long-lived proteins, protein complexes, and damaged organelles in cells. So we first used the Atg7 siRNA plasmid to inhibit the expression of autophagy-related protein Atg7. As seen in Fig. 7a, b, c, d, siRNA Atg7 plasmid can decrease the expression of Atg7 in U87 and U251 cells. 3-MA is used to inhibit autophagy and study the involved mechanism of autophagy (lysosomal self-degradation) and apoptosis under various conditions. 3-MA inhibits autophagy by blocking autophagosome formation via the inhibition of type III Phosphatidylinositol 3-kinases (PI-3K). We used the autophagy marker protein LC3 to detect the effect of Atg7 siRNA and autophagy inhibitor 3-MA on autophagy induced by bortezomib. As shown in Fig. 7e, f, g, h, Atg7 siRNA and autophagy inhibitor 3-MA decreased the expression of LC3-II. These results indicated that Atg7 siRNA and 3-MA can inhibit autophagy induced by bortezomib in U87 and U251 cells.

### Inhibition of autophagy enhances apoptosis induced by bortezomib in U87 and U251 cells

**Fig. 8:**
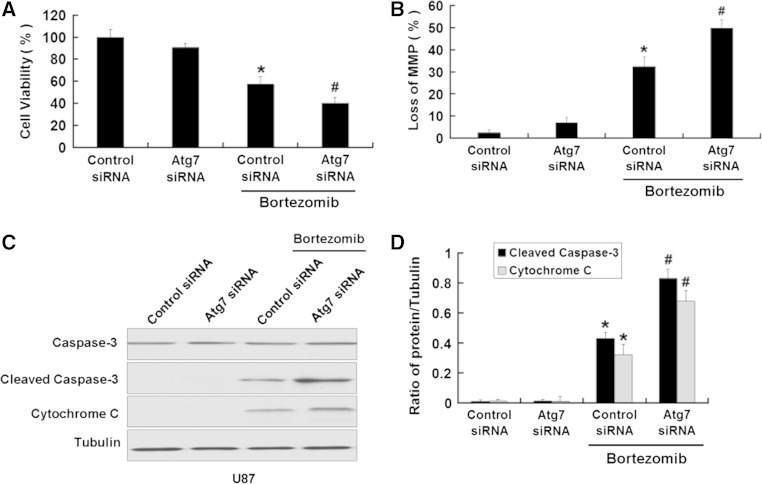
Inhibition of autophagy by Atg7 siRNA enhances apoptosis induced by bortezomib in U87 cells. **a** U87 cells were treated with control siRNA or Atg7 siRNA with or without bortezomib (10 nM, 24 h). Cells viability was determined by MTT assay. Data are presented as mean ± SD, *n* = 6. **b** U87 cells treated with control siRNA or Atg7 siRNA with or without bortezomib (10 nM, 24 h). The cells were harvested after treatment and were stained with JC-1. **c** Western blot analysis for the expressions of Caspase-3, Cleaved caspase-3, and Cytochrome C in U87 cells treated with control siRNA or Atg7 siRNA with or without bortezomib (10 nM, 24 h). **d** Quantitation of Cleaved caspase-3 and Cytochrome C levels in U87 cells treated with control siRNA or Atg7 siRNA with or without bortezomib. Data are presented as mean ± SD, *n* = 3, *P < 0.05 versus control group. ^**#**^ *P* < 0.05 versus bortezomib group

**Fig. 9:**
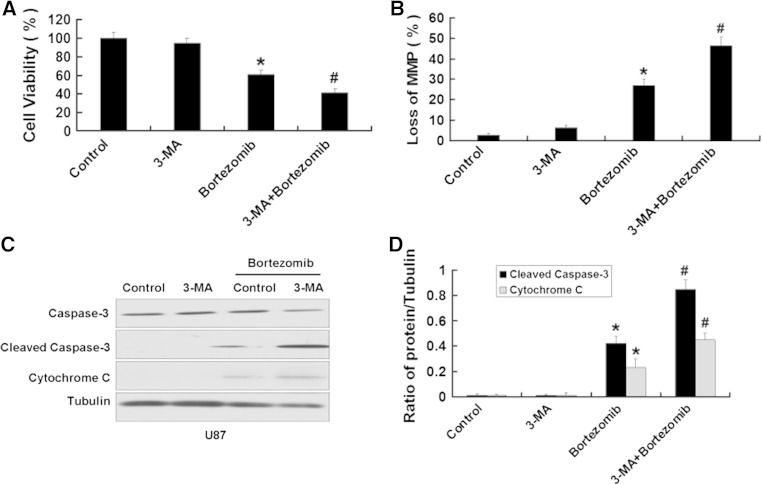
Inhibition of autophagy by autophagy inhibitor 3-MA enhances apoptosis induced by bortezomib in U87 cells. **a** U87 cells were treated with control (DMSO) or 3-MA (5 mM) with or without bortezomib (10 nM) for 24 h. Cells viability was determined by MTT assay. Data are presented as mean ± SD, *n* = 6. **b** U87 cells treated with control (DMSO) or 3-MA (5 mM) with or without bortezomib (10 nM) for 24 h. The cells were harvested after treatment and were stained with JC-1. **c** Western blot analysis for the expressions of Caspase-3, Cleaved caspase-3, and Cytochrome C in U87 cells treated with control (DMSO) or 3-MA (5 mM) with or without bortezomib (10 nM) for 24 h. **d** Quantitation of Cleaved caspase-3 and Cytochrome C levels in U87 cells treated with control (DMSO) or 3-MA (5 mM) with or without bortezomib (10 nM) for 24 h. Data are presented as mean ± SD, *n* = 3, *P < 0.05 versus control group. ^**#**^ *P* < 0.05 versus bortezomib group

**Fig. 10:**
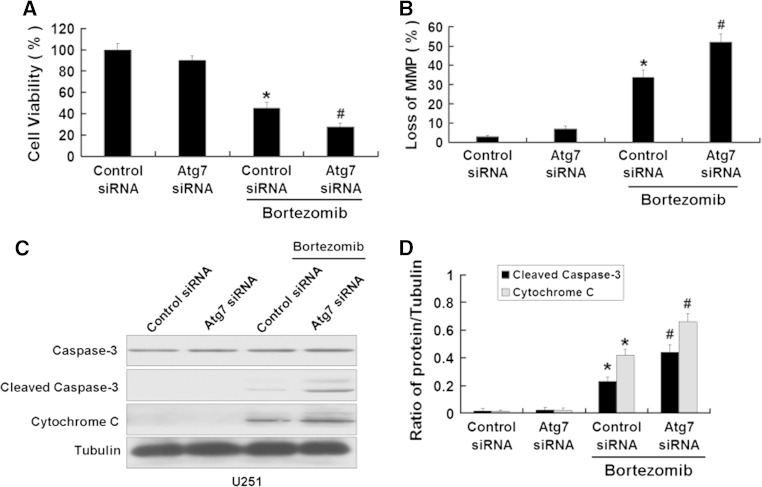
Inhibition of autophagy by Atg7 siRNA enhances apoptosis induced by bortezomib in U251 cells. **a** U251 cells were treated with control siRNA or Atg7 siRNA with or without bortezomib (10 nM, 24 h). Cells viability was determined by MTT assay. Data are presented as mean ± SD, *n* = 6. **b** U251 cells treated with control siRNA or Atg7 siRNA with or without bortezomib (10 nM, 24 h). The cells were harvested after treatment and were stained with JC-1. **c** Western blot analysis for the expressions of Caspase-3, Cleaved caspase-3, and Cytochrome C in U251 cells treated with control siRNA or Atg7 siRNA with or without bortezomib (10 nM, 24 h). **d** Quantitation of Cleaved caspase-3 and Cytochrome C levels in U251 cells treated with control siRNA or Atg7 siRNA with or without bortezomib. Data are presented as mean ± SD, *n* = 3, *P < 0.05 versus control group. ^**#**^ *P* < 0.05 versus bortezomib group

**Fig. 11:**
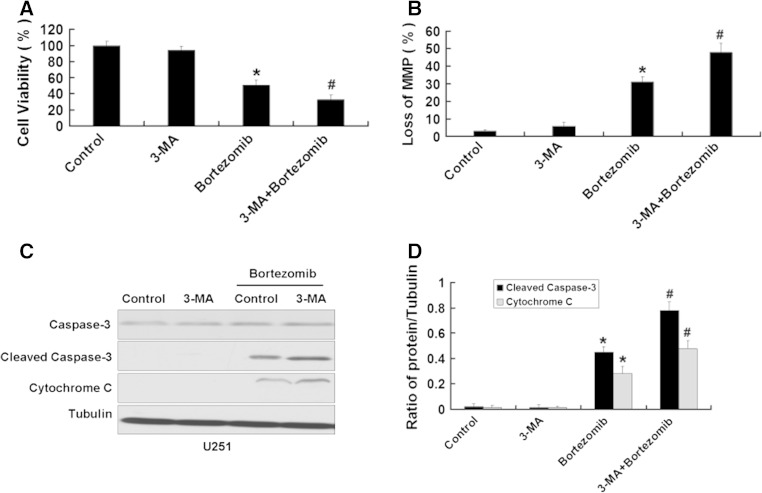
Inhibition of autophagy by autophagy inhibitor 3-MA enhances apoptosis induced by bortezomib in U251 cells. **a** U251 cells were treated with control (DMSO) or 3-MA (5 mM) with or without bortezomib (10 nM) for 24 h. Cells viability was determined by MTT assay. Data are presented as mean ± SD, *n* = 6. **b** U251 cells treated with control (DMSO) or 3-MA (5 mM) with or without bortezomib (10 nM) for 24 h. The cells were harvested after treatment and were stained with JC-1. **c** Western blot analysis for the expressions of Caspase-3, Cleaved caspase-3, and Cytochrome C in U251 cells treated with control (DMSO) or 3-MA (5 mM) with or without bortezomib (10 nM) for 24 h. **d** Quantitation of Cleaved caspase-3 and Cytochrome C levels in U251 cells treated with control (DMSO) or 3-MA (5 mM) with or without bortezomib (10 nM) for 24 h. Data are presented as mean ± SD, *n* = 3, *P < 0.05 versus control group. ^**#**^ *P* < 0.05 versus bortezomib group

We further used the above method to inhibit the autophagy induced by bortezomib inhibition of autophagy. First, we used MTT assay to detect the viability of U87 cells treated by bortezomib with or without Atg7 siRNA. As shown in Fig. 8a, b, Atg7 siRNA intensified the growth inhibition of U87 cells and increased the loss of the MMP in U87 cells induced by bortezomib. We next detected the apoptotic-related proteins cleaved caspase-3 and cytochrome C. As seen in Fig. 8c, d, Atg7 siRNA further increased the expression of apoptotic-related proteins cleaved caspase-3 and cytochrome C induced by bortezomib. Meanwhile, we used the autophagy inhibitor 3-MA to further explore the role of autophagy in apoptosis induced by bortezomib. As shown in Fig. 9a, 3-MA can further decrease the viability of U87 cells induced by bortezomib. At the same time, 3-MA enhanced the expression of apoptotic related proteins cleaved caspase-3, cytochrome C and the loss of MMP in U87 cells (Fig. 9b, c, d). The similar results can be seen in another human glioblastoma cell line U251 (Figs. 10 , 11). These results showed that inhibition of autophagy can increase apoptosis induced by bortezomib in human glioblastoma cells.

## Discussion

Recent studies have shown that proteasome inhibitors serve as a new and promising class of anticancer agents [27–31]. In this study, we showed that the proliferation of U251 and U87 cells was inhibited by bortezomib in a dose-dependent manner. The results also illustrated that bortezomib treatment causes U251 and U87 cells to undergo apoptosis in a dose-dependent manner, as evidenced by activation of the apoptotic markers cleaved caspase 3 and cytochrome C, may be as the mechanism of the growth inhibition. Moreover, we also found that autophagy was activated in U251 and U87 cells after they were incubated with bortezomib.

However, some articles showed that single use of proteasome inhibitors may not be effective for resistant myeloma and solid malignants [32, 33]. A number of regimes have been demonstrated that autophagy suppression enhanced cancer cell death [34–36]. Although cell death resulting from progressive cellular consumption has been attributed to unrestrained autophagy, leading to the notion that autophagy is a nonapoptotic form of programmed cell death, most evidence supports autophagy as a survival pathway required for cellular viability [37].

As the amount of LC3 protein, especially LC3-II, correlates with the extent of autophagy, the effects of bortezomib and 3-MA (an autophagy inhibitor) or the siRNA of Atg7 (the essential autophagy gene) on LC3 protein expressions were studied. Therefore, to determine whether the autophagy induced by bortezomib would be blocked by 3-MA or the siRNA of Atg7 in U251 and U87 cells, we co-treated the U251 and U87 cells with bortezomib and 3-MA or Atg7 siRNA. The results showed that both 3-MA and Atg7 siRNA effectively blocked autophagy induced by bortezomib, as evidenced by autophagy markers. Furthermore, to determine whether the antitumor effects of bortezomib are enhanced by the modulation of autophagy, U251 and U87 cells were also treated with the combination of bortezomib and 3-MA or Atg7 siRNA. Notably, bortezomib alone had an inhibitory effect; but the combination of bortezomib and 3-MA or Atg7 siRNA led to a further inhibition of U251 and U87 cells proliferation, indicating that suppression of the proteasome together with the autophagy inhibitor caused more potent cells apoptosis. Further results showed that combined treatment of bortezomib and 3-MA or siRNA knockdown of Atg7 resulted in a marked increase in caspase 3 activation compared with the tumor cells that were treated with bortezomib alone. These findings indicated that activation of autophagy in glioblastoma cells directly contributes to the survival of cancer cells treated with proteasome inhibitors.

In conclusion, we provided evidence that bortezomib, the proteasome inhibitor, exerts growth inhibition in human U251 and U87 cells in vitro, and suppression of the proteasome increases autophagy. Moreover, we also demonstrated that 3-MA or Atg7 siRNA blocked the autophagy induced by bortezomib and the combined use of bortezomib and 3-MA or Atg7 siRNA enhanced cell death in human U251 and U87 cells. The study offers a novel strategy for enhancing cell growth inhibition in glioblastoma cells. Combination treatment of the proteasome and autophagy inhibitors may be a novel modality for treating glioblastoma.
